# Deciphering the genetic basis for polyketide variation among mycobacteria producing mycolactones

**DOI:** 10.1186/1471-2164-9-462

**Published:** 2008-10-07

**Authors:** Sacha J Pidot, Hui Hong, Torsten Seemann, Jessica L Porter, Marcus J Yip, Artem Men, Matthew Johnson, Peter Wilson, John K Davies, Peter F Leadlay, Timothy P Stinear

**Affiliations:** 1 Department of Microbiology, Monash University, Clayton, 3800, Australia; 2 Department of Biochemistry, University of Cambridge, Cambridge, CB21EW, UK,; 3 Victorian Bioinformatics Consortium, Monash University, Clayton, 3800, Australia; 4 Australian Genome Research Facility, University of Queensland, St Lucia, 4072, Australia

## Abstract

**Background:**

Mycolactones are immunosuppressive and cytotoxic polyketides, comprising five naturally occurring structural variants (named A/B, C, D, E and F), produced by different species of very closely related mycobacteria including the human pathogen, *Mycobacterium ulcerans*. In *M. ulcerans *strain Agy99, mycolactone A/B is produced by three highly homologous type I polyketide megasynthases (PKS), whose genes (*mlsA1*: 51 kb, *mlsA2*: 7.2 kb and *mlsB*: 42 kb) are found on a 174 kb plasmid, known as pMUM001.

**Results:**

We report here comparative genomic analysis of pMUM001, the complete DNA sequence of a 190 kb megaplasmid (pMUM002) from *Mycobacterium liflandii *128FXT and partial sequence of two additional pMUM replicons, combined with liquid chromatography-tandem mass spectrometric (LC-MS/MS) analysis. These data reveal how PKS module and domain differences affecting MlsB correlate with the production of mycolactones E and F. For mycolactone E these differences from MlsB in *M. ulcerans *Agy99 include replacement of the AT domain of the loading module (acetate to propionate) and the absence of an entire extension module. For mycolactone F there is also a reduction of one extension module but also a swap of ketoreductase domains that explains the characteristic stereochemistry of the two terminal side-chain hydroxyls, an arrangement unique to mycolactone F

**Conclusion:**

The mycolactone PKS locus on pMUM002 revealed the same large, three-gene structure and extraordinary pattern of near-identical PKS domain sequence repetition as observed in pMUM001 with greater than 98.5% nucleotide identity among domains of the same function. Intra- and inter-strain comparisons suggest that the extreme sequence homogeneity seen among the mls PKS genes is caused by frequent recombination-mediated domain replacement. This work has shed light on the evolution of mycolactone biosynthesis among an unusual group of mycobacteria and highlights the potential of the *mls *locus to become a toolbox for combinatorial PKS biochemistry.

## Background

**Figure 1:**
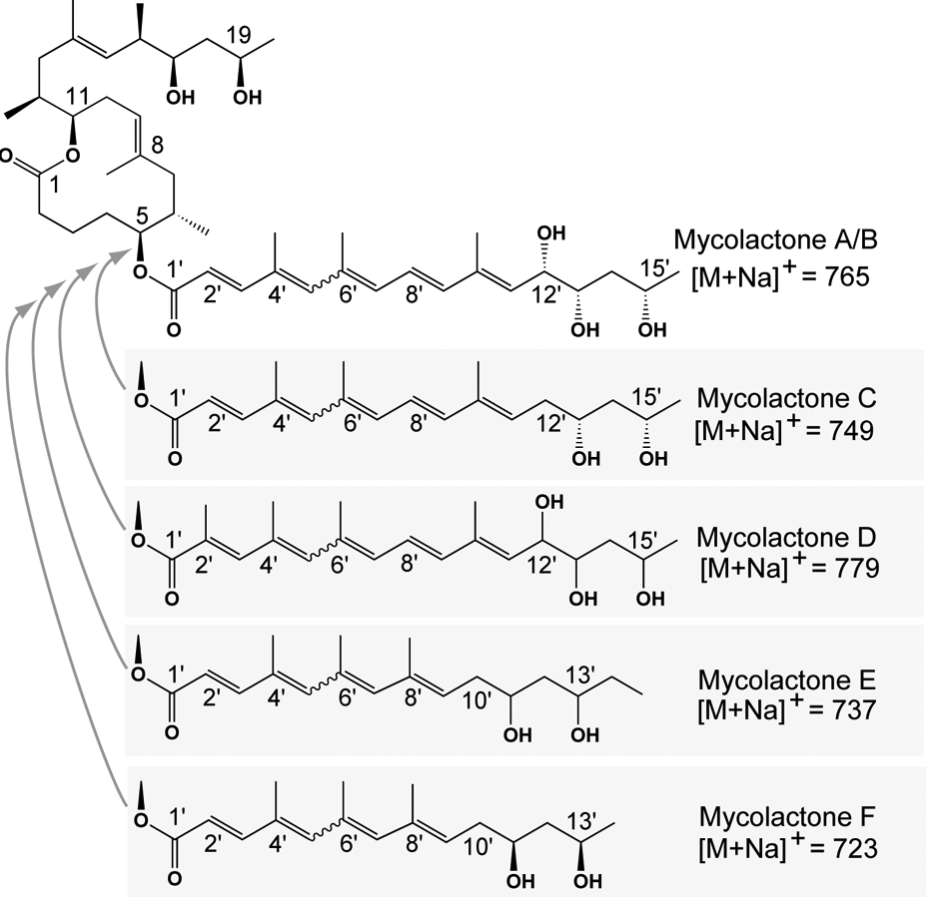
Structures of the mycolactones identified to date.

Mycolactone is a polyketide-derived, secondary metabolite and a major virulence factor of the human pathogen *Mycobacterium ulcerans *(MU), the causative agent of Buruli ulcer. At picogram concentrations mycolactone has immunosuppressive properties and at higher concentrations it is cytotoxic for mammalian cells [1]. The molecule is composed of an invariant core comprising a 12-membered macrolactone and side-chain that is esterified to a highly unsaturated acyl side chain, the latter structure varying amongst different MU strains (Figure 1) [1]. MU strains from Africa, Australia and China produce variants named mycolactones A/B, C, and D, respectively whilst *Mycobacterium liflandii *(ML), a pathogen of frogs, produces mycolactone E, and the fish pathogens (*Mycobacterium pseudoshottsii *and *Mycobacterium marinum *DL240490 (DL) and others) produce mycolactone F [2-9] (Figure 1). Despite the multiple species names given to mycolactone-producing mycobacteria (MPM), multi locus sequence analysis (MLSA) of all these strains indicates they share greater than 98% nucleotide identity [10]. The MPM appear to have evolved from a common *M. marinum *ancestor by acquisition of a large circular plasmid that conferred the ability to make mycolactones and then spread throughout the world, occupying different hosts [10-12].

**Figure 2:**
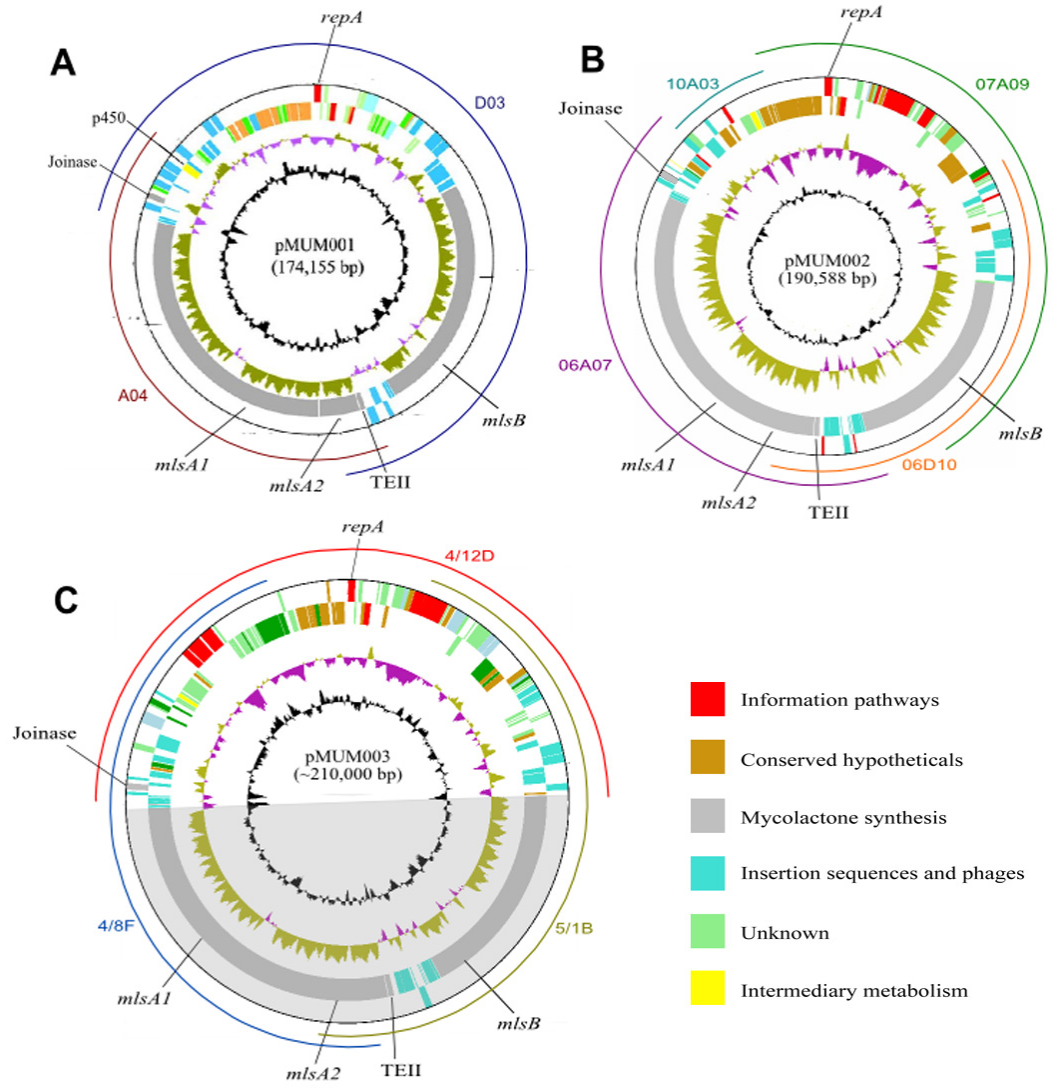
**Circular maps of A) pMUM001, B) pMUM002 and C) pMUM003**. The innermost black circle represents G+C content, and the next circle outwards shows GC skew (G-C)/(G+C) over a 1 kb range. Moving outwards, the next two circles represent reverse and forward strand protein coding DNA sequences, respectively, which are coloured by functional class. Mapped onto pMUM002 and pMUM003 are the location and size of each of the BACs used for sequencing of these plasmids. The shading of pMUM003 indicates that the *mls *genes of pMUM003 were not completely sequenced.

In MU strain Agy99, the only strain for which a genome sequence is currently available, a 174 kb megaplasmid named pMUM001 has three very large genes (*mlsA1*: 51 kb, *mlsA2*: 7 kb and *mlsB*: 42 kb) (Figure 2A) [11] that encode the modular type I PKSs required for mycolactone synthesis. The plasmid also has three putative accessory genes (MUP038, encoding a type II thioesterase; MUP045 encoding a beta-ketoacyl synthase and *cyp140A7 *[MUP053] encoding a cytochrome P450 hydroxylase). MlsA1 and MlsA2 form a nine-extension module complex that synthesises the mycolactone core, whilst MlsB is a single polypeptide, comprising seven extension modules that are required for the synthesis of the side chain.

Bacterial type I PKS are modular multi-enzymes and act as molecular assembly lines for the formation of polyketides [13]. These enzymes function in a sequential manner where each PKS module is responsible for one round of chain elongation via the addition of (usually) either acetate or propionate, supplied to the PKS as an activated malonyl or methylmalonyl-CoA thioester. Within each PKS module are a series of covalently linked enzymatic domains that process the growing polyketide chain before passing it downstream to the next module in the system [14]. The minimal set of enzymatic domains required for PKS activity includes ketosynthase (KS), acyltransferase (AT) and an acyl carrier protein (ACP) domain [15]. Ketoreductase (KR), dehydratase (DH) and enoylreductase (ER) domains are also commonly found in modules and form a so-called reductive loop, providing reducing enzyme activities that modify the two or three-carbon unit being added to the polyketide [15].

The mycolactone PKS (Mls locus) exhibits a number of unusual features that distinguish it from other type I PKS complexes. Firstly, the Mls PKSs are exceptionally large with a total predicted monomeric size of ~3.0 MDa, placing them amongst the largest known cellular enzymes [11]. Secondly, there is an unprecedented level of genetic identity amongst the enzymatic domains of all Mls modules. For other type I PKSs, functionally identical domains from the same PKS generally share 40 – 70% amino acid (aa) sequence identity [16], however, identity between domains of the Mls locus ranges from 98.7 – 100% [11].

The extreme sequence homology within the Mls locus might be expected to provide a rich substrate for homologous recombination. Indeed, mycolactone negative mutants frequently arise among laboratory passaged MU strains; caused by partial deletion of the *mls *genes [17]. Mycolactone D produced by an MU strain from China differs from mycolactone A/B by the substitution of a methylene at C2' of the acyl side chain (Figure 1). In this strain, the final extension module of MlsB possesses an AT domain with propionate rather than acetate specificity, suggesting natural recombination within *mlsB *[18]. However, mycolactone structural variations are quite restricted. MPM recovered from around the world for 70 years all produce mycolactones with an absolutely conserved core and all variations occur within the fatty-acyl side-chain (Figure 1). The role mycolactones play in the survival of MPM and why variation is only tolerated (or provides a selective advantage) in the side-chain is unknown.

In this study we investigated the genetic basis for production of these variant structures, in particular for the frog pathogen *M. liflandii*. We determined the complete DNA sequence of the 190 kb megaplasmid (pMUM002) from *Mycobacterium liflandii *128FXT and the partial sequence of pMUM replicons from *Mycobacterium marinum *DL240490 and *M. ulcerans *Japan 753. We also employed LC-MS/MS structural analysis of their respective mycolactones. Our results show that mycolactones produced by different MPM are caused by genetic rearrangements between homologous PKS domains of the Mls locus, highlighting the plasticity of this region and its potential for combinatorial polyketide biochemistry.

## Results

### Overview of pMUM002 from *M. liflandii *128FXT

Assembly of the complete DNA sequence of pMUM002 from four overlapping BAC clones (06A07: 77 kb, 06D10: 110 kb, 07A09: 90 kb, 10A03: 15 kb), revealed a 190,588 bp circular element containing 95 predicted CDS. A summary of the main features of pMUM002 compared with pMUM001 is shown in Table 1 and an overview of its CDS distribution is shown in Figure 2B.

**Table 1:** Comparison of general features of three pMUM plasmids

	pMUM001	pMUM002	pMUM003
	
Size (bp)	174155	190588	Predicted 210 kb
No. genes involved in mycolactone synthesis	6	5	5
Mycolactone related genes kb (% of plasmid)	105 (60%)	97.4 (51%)	97.4 (48%)*
Non-mycolactone related DNA kb (% of plasmid)	69.2 (40%)	93.2 (49%)	104.5 (52%)*
Pseudogenes	7	22	21

* percentage based on predicted size of plasmid

Plasmid pMUM002 encodes 61 of its CDS (63.5%) on the reverse strand and has a G+C content of 62.9%, which is similar to pMUM001 (62.7% GC) [19]. Five genes, spanning 97.2 kb (51%) of the plasmid, are predicted to be involved in mycolactone biosynthesis, with the same arrangement as those present on pMUM001 [11]. These genes include the three type I polyketide synthases (*mlsA1*: 51,060 bp, *mlsA2*: 7,233 bp and *mlsB*: 37,059 bp), a type II thioesterase (MULP_063) and a FabH-like type III ketosynthase (MULP_070). Sequencing of the plasmid confirmed the previously reported absence of the cytochrome P450 hydroxylase gene *cyp140A7 *[7], which is responsible for the production of mycolactones A/B and D via hydroxylation at the C'12 position of the acyl side chain of these variants [17].

### Overview of pMUM003 from *M. marinum *DL240490

Three BAC clones from a DL genomic library were predicted to span all of pMUM003 (04D12: 110 kb, 051B: 99 kb, 048F: 98 kb) and these clones were selected for further analyses. Based on the sizes of these BACs as determined by pulsed field gel electrophoresis (PFGE) and the results of end-sequencing, a map of pMUM003 was constructed indicating it is a circular replicon with an estimated size of ~210 kb (Figure 2C). Due to the complex and time-consuming nature of sequencing mycolactone PKS loci caused by the extreme sequence repetition, only the non-PKS region of pMUM003 was fully sequenced for this study. The BAC clone 0412D spanned this region of the plasmid from the 5' end of *mlsB *to the 3' end of *mlsA1 *(Figure 2C) and the complete sequence of 0412D was 104,530 bp with 102 predicted CDS. The two other selected BAC clones (051B and 048F) were used in PCR, sequencing and Southern blot analyses to confirm the domain and module composition of specific regions of the pMUM003 *mls *genes (see below).

### Comparison of pMUM plasmids confirms their common origin

Both pMUM002 and pMUM003 share the same overall organization as pMUM001 with a core set of 42 CDS common to all three plasmids (see Additional file 1). A complete list of CDS from pMUM002 and the non-PKS region of pMUM003 is presented and discussed in Additional files 2 and 3. The pMUM001 gene MUP045 encodes a putative FabH-like type III ketosynthase gene that is essential for mycolactone production [20] and may have acyltransferase activity, linking the mycolactone core and acyl-side chain. This gene is present in all three plasmids (MUP045, MULP_070, MUDP_005) and they share very high sequence identity; their predicted protein products differing by only one amino acid, suggesting the importance of the gene for mycolactone biosynthesis. There were also several regions of difference observed between the plasmids that included deletions, insertions and other genetic rearrangements. These alterations are presumably mediated by the many ISE found in all pMUM plasmids. For example, the absence of *cyp140A7 *in pMUM002 & 3, encoding a p450 hydroxylase that modifies mycolactone, is most likely explained by an IS*2606*-mediated deletion (see Additional file 1). Overall, it appears that the main function of the pMUM plasmids is for the production of mycolactones with no other obvious virulence or virulence-associated genes present.

### The pMUM002 Mls module and domain arrangements correspond with the structure of mycolactone E

**Figure 3:**
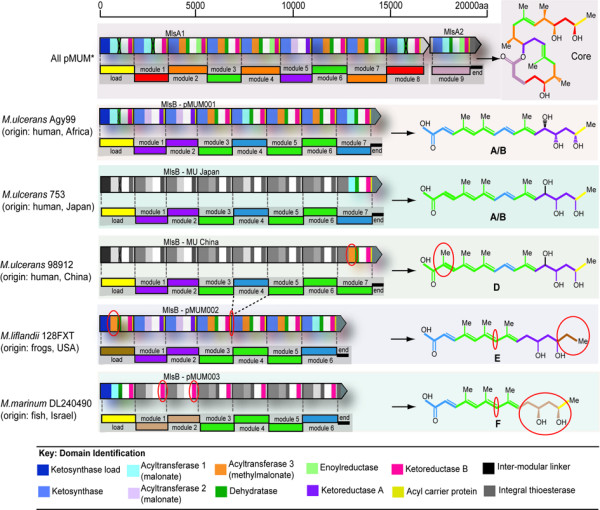
**Genetic organisation of the mycolactone biosynthetic cluster from pMUM001, pMUM002 and proposed organization for pMUM003**. Mycolactone PKS module and domain structure is outlined, with the figure key showing the type of domains present in each of the modules. The mycolactone modules are colour coded based on the type of module responsible for the addition of each two-carbon unit. Shaded modules indicate that the DNA sequence of these regions is unknown or not yet confirmed. *Organisation of *mlsA1 *and *mlsA2 *for all pMUM examined to date, based upon toxin structures.

The complete sequencing of the *mls *locus of pMUM002 from ML revealed an extraordinarily high degree of similarity to the *mls *locus of pMUM001 with a module and domain arrangement in near-perfect agreement with predictions based on the proposed structure of mycolactone E [21]. The core macrocyclic lactone of mycolactone E is synthesised by MlsA1 and MlsA2, comprising a loading module and nine extension modules, terminating in a putative integral C-terminal thioesterase (Figure 3). Similarly the acyl-side chain is synthesised by MlsB and comprises a load module with six extension modules (Figure 3). MlsB from pMUM002 is 12,353 aa, which is 1,778 aa shorter than MlsB from pMUM001 (14,131 aa). The size difference is due to the absence in pMUM002 of the equivalent of module 4 from pMUM001, an absence that corresponds precisely with the absence of a CH = CH moiety in the acyl-side chain of mycolactone E [21] (Figure 3). There was also perfect agreement between observed structure and the pattern of acetate and propionate incorporation predicted from sequence analysis of the AT domains. In particular, the AT domain of the MlsB load module is methylmalonyl-CoA-specific (AT-III, Figure 3, see Additional file 4), indicating that ML uses a propionate starter unit which corresponds perfectly with structure-based predictions [21]. These results provide another example of a "swappable" domain location as the equivalent AT domain in pMUM001 uses an acetate starter unit (AT-II). The oxidation state predicted from the Mls sequence after each stage of chain extension also aligns very closely with mycolactone E structure. However, like pMUM001, extension module 2 of MlsA1 contains apparently inactive DH and ER domains [11].

**Figure 4:**
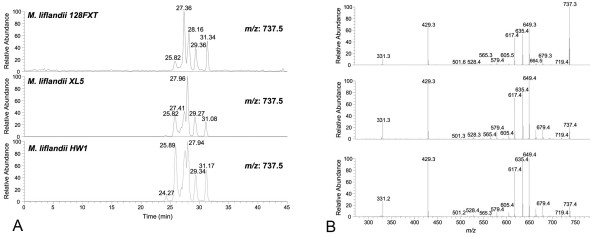
**LC-MS/MS analysis of lipid extracts from three different ML strains**. A) Ion traces for *m*/*z *737 ([M+Na]^+^). B) MS/MS spectra of ion at *m*/*z *737.

To reinforce the veracity of the above findings and to resolve an outstanding discrepancy regarding the correct structure of mycolactone E [8,21], LC-MS/MS analysis was used to analyse mycolactones detected in lipid extracts from two additional ML strains (ML XL5 and ML HW1). These analyses revealed an identical ion trace and fragmentation pattern for *m/z *737 ([M+Na]^+ ^ion) as reported for ML 128FXT (Figure 4). Thus, the combination of these concordant structural data from multiple ML strains together with the sequence analysis of the *mls *genes from ML 128FXT provide compelling evidence that the mycolactone structure first reported by Hong *et al *[21] is correct.

### Alterations in pMUM003 MlsB module and domain arrangement correspond with the structure of mycolactone F

Although the complete sequence of the *mls *locus from pMUM003 was not determined in this study, the two BAC clones 048F and 051B that spanned *mlsA *and *mlsB *respectively (Figure 2C) permitted some investigations into the genetic basis for the production of mycolactone F from DL. Using BAC clone 051B as template, PCR and sequencing of the load module of *mlsB *identified that this module has an AT-I (malonate) domain, indicating an acetate starter unit for mycolactone F side-chain synthesis. This arrangement, which is supported by structural data [5,8] is the same as pMUM001 but different to ML which has an AT-III (methylmalonate) domain (Figure 3).

**Figure 5:**
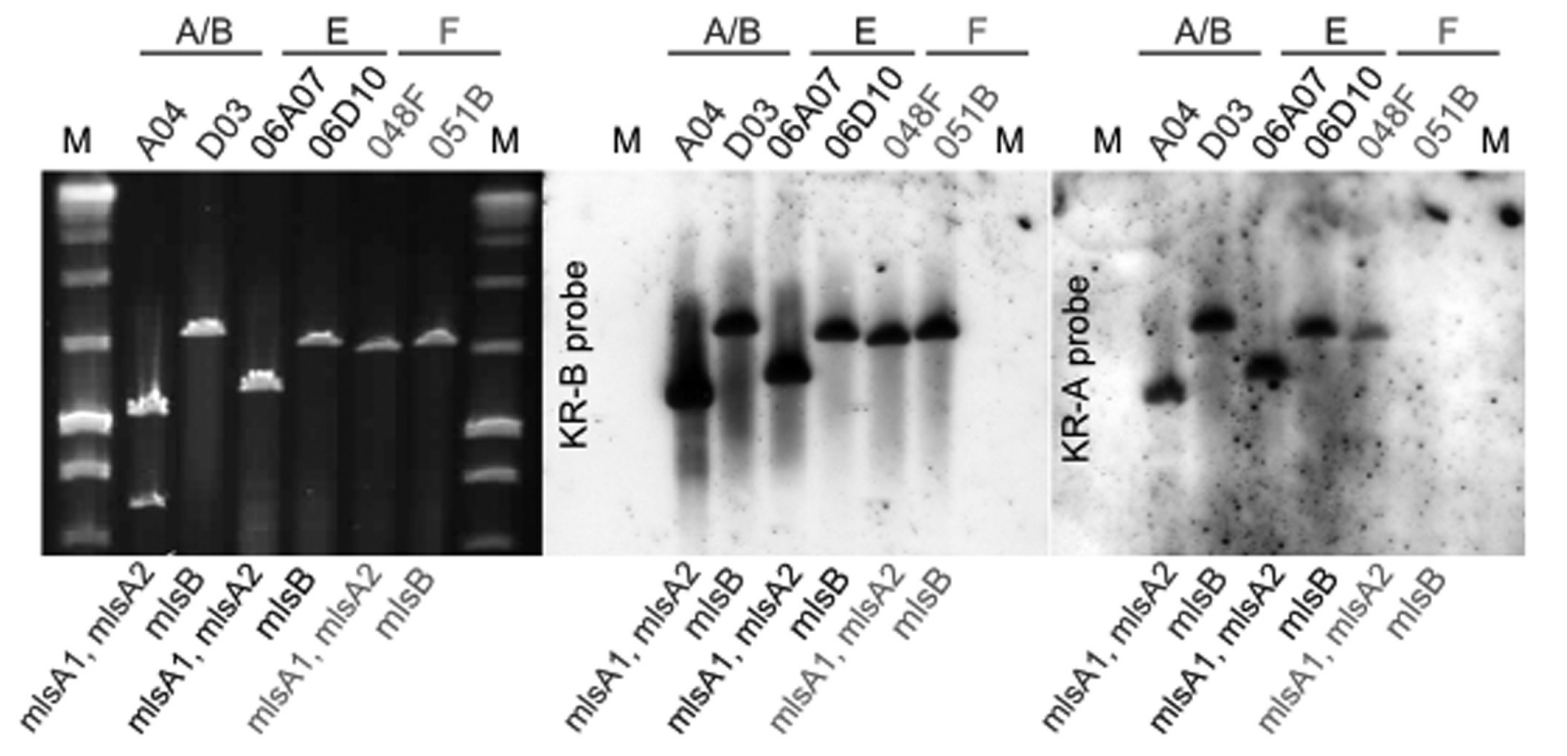
**Pulsed-field gel electrophoresis and Southern hybridisation analysis of pMUM001, pMUM002 and pMUM003**. A) Pulsed-field gel electrophoresis of *Dra*I digested BACs containing *mlsA *or *mlsB *from pMUM001 (A04 and D03, respectively), pMUM002 (06A07 and 06D10, respectively), and pMUM003 (048F and 051B, respecitvley). B) Southern hybridisation with probe for KR-B domain. C) Southern hybridisation with probe for KR-A domain.

The recent total synthesis of mycolactone F showed that the hydroxyl groups at C11' and C13' of its acyl side chain have the opposite stereochemistry to other mycolactones [5]. KR domains control the geometry of the hydroxyls and two types of this domain have been noted (A-type and B-type), with each type responsible for a specific stereochemistry [22,23]. As the C11' and C13' side-chain hydroxyl arrangement reported for mycolactone F had not been observed in other mycolactones, it suggested that a switch from A-type to B-type KR domains had occurred within the first two extension modules of MlsB from pMUM003. To test this hypothesis, we performed Southern hybridisation analysis of BACs containing either *mlsA *or *mlsB *from pMUM001, pMUM002 and pMUM003 with probes for either the KR-A or KR-B domain, and as predicted, Southern analysis confirmed that there are no A-type KR domains in *mlsB *of pMUM003 (Figure 5), consistent with our hypothesis that the A-type KR domains have been replaced. This result also suggests the presence of a previously unidentified Mls module type consisting of a combination of KS, AT-II (malonate) and B-type KR domains (Figure 3).

### MLSB module 7 is different between MU strains from China and Japan

**Figure 6:**
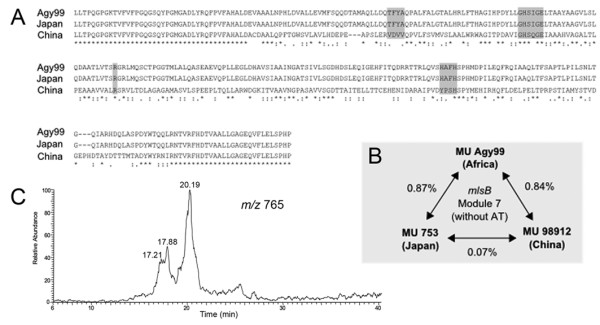
**MU Japan makes mycolactone A/B not D**. A) Amino acid sequence comparisons of AT domains of Module 7 of MU Agy99, MU China 98912 and MU Japan 753. Shaded residues are those known to be essential for AT substrate specificity. B) Percentage difference over 4135 bp between the three *mlsB *Module 7 sequences, exclusive of the AT domain of each module. C) Ion trace of *m*/*z *765 from LC-MS/MS analysis of lipid extracts from MU Japan 753.

The above examples of module and domain rearrangement prompted a closer inspection of the mycolactones produced by MU strains from Japan and China. MU strains from these countries are genetically very closely related with only one discriminating allele by MLSA and identical InDel patterns [10,24]. Previous investigations had revealed that strain MU98912 from China makes a modified mycolactone (mycolactone D) due to the presence of an AT-III (methylmalonate) domain in extension module 7 of MlsB [18]. However, the only published report of the structure of mycolactones from Japan suggested that Japanese strains made mycolactone A/B [6] and not mycolactone D as might have been expected. We used PCR and sequencing of *mlsB *module 7 from the Japanese MU strain 753 to check the domain arrangement and found that it has an AT-II (malonate) domain (Figure 6), which is different to that of MU98912 China but which is the expected arrangement for mycolactone A/B synthesis (Figure 3). Analysis of lipid extracts from MU753 Japan by LC-MS/MS confirmed that this strain does make mycolactone A/B, not mycolactone D (Figure 6) [18]. Sequence comparisons of this region from MU753 with the same region among MU98912 and MUAgy99 Africa shows that module arrangements and the corresponding mycolactone produced does not necessarily correlate with strain relatedness (Figure 6), indicating that remodelling of the *mls *genes is occurring at a frequency independent of single nucleotide mutation and highlighting the dynamic nature of this locus.

### Ongoing replacement, duplication and deletion of the highly conserved Mls modules and domains

**Figure 7:**
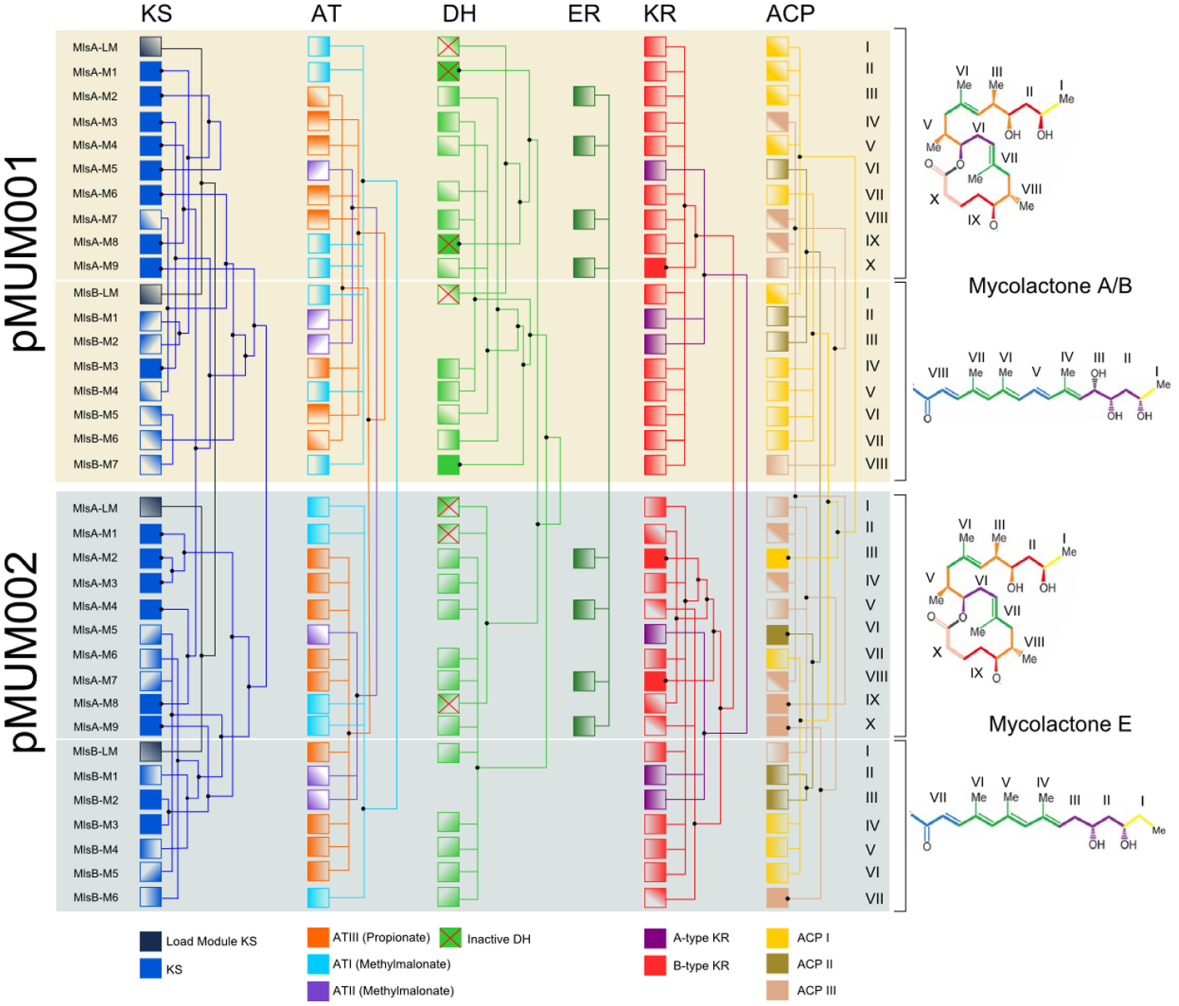
**Modified neighbour-joining tree showing the relatedness of the PKS domains of the mycolactone PKS produced by pMUM001 and pMUM002**. Identical shading patterns amongst domains shows identical DNA sequence, with the exception of solid colour blocks, which represent domains with individual DNA sequences. Each module is labelled with a roman numeral, corresponding to the step of either mycolactone core or side-chain biosynthesis for which it is responsible.

As observed in pMUM001, the pMUM002 *mls *DNA is highly repetitive and exhibits extreme sequence conservation between domains with an identical catalytic function. For example, the 15 KS domains share > 99.1% nucleotide identity over 1257 nts, which translates to only nine variable amino acid residues among 419 aa. There are three types of domains (LM-KS, AT-I and AT-II) that have 100% intra-species nucleotide identity within both pMUM001 and pMUM002. The extreme sequence identity is also conserved between strains and falls only marginally to 99.2 – 99.8% for these domains (Table 2). The most variable of the enzymatic domains between species are the ACP-I domains (95.2% nucleotide identity). When modules of identical domain arrangement were compared, nucleotide identity ranged from 98.6% (*mlsA *modules 2, 4 and 7) to 99.9% (*mlsA *module 5 and *mlsB *modules 1 and 2). Phylogenetic analysis of the domains and modules across pMUM001 and pMUM002 emphasise the high degree of relatedness between these genetic loci but also show that they cluster by strain, suggesting that evolution of the locus is occurring vertically rather than via horizontal exchanges between strains (Figure 7).

**Table 2:** Percentage nucleotide (nts) and amino acid (aa) identity amongst domains of the mycolactone PKS from pMUM001 and pMUM002

	**pMUM001**		**pMUM002**		**Interspecies**	
**Domain **(nts/aa)	nts	aa	nts	aa	nts	aa

**Ketosynthase-load **(1149/383)	100	100	100	100	99.6	98.7

**Ketosynthase **(1260/419)	98.9	97.3	99.1	97.8	98.6	96.7

**Acyltransferase-I **(1056/352)	100	100	100	100	99.8	99.7

**Acyltransferase-II **(999/333)	100	100	100	100	99.2	99.1

**Acyltransferase-III **(1056/352)	99.9	100	100	100	99.8	99.7

**Dehydratase **(465/155)	98.7	96.1	99.3	99.3	98.3	98.7

**Enoylreductase **(948/316)	100	100	100	100	100	100

**Ketoreductase A **(528/176)	100	100	100	100	100	100

**Ketoreductase B **(540/180)	99.6	98.9	99.4	98.3	98.7	97.2

**Acyl carrier protein-I **(210/70)	95.7	95.7	98.6	100	95.2	95.7

**Acyl carrier protein-II **(210/70)	100	100	99.5	98.6	99.1	97.1

**Acyl carrier protein-III **(210/70)	98.6	98.6	97.6	95.7	96.2	94.3

**Chromosomal**					99.6*	99.4

**Plasmid (other)**					99.7	99.3

* Comparisons of plasmid and chromosomal sequences are taken from mulit-locus sequence analysis of concatenated DNA sequences of 3210 nts and 1070 nts respectively [6].

The previously reported domain swap in MlsB of MU98912 China suggested either replacement of domains, combinations of domains, or entire modules, possibly by a gene conversion mechanism. A detailed comparison of modules with an identical domain configuration revealed that identity between one or more domains does not equate with identity over the whole module (see Figure 7). For example, when an interspecies nucleotide comparison is performed across the region from the AT-II domain to the KR-A domain of module 5 of *mlsA *and modules 1 and 2 of *mlsB*, there is 100% nucleotide identity over 2682 nts. However, the KS and ACP domains of these modules have only 99.2% and 99.5% identity, respectively. When other modules of identical organisation are analysed in this manner, similar patterns are seen (Figure 7). Another example of this is the presence of an active DH domain in the LM of *mlsB *of pMUM002, which follows an AT-III domain. In pMUM001, the two domains seen in these positions are a DH domain, with a predicted inactive catalytic site, and an AT-I domain (Figure 3 and Figure 7). In no part of *mlsA1*, *mlsA2 *or *mlsB *of pMUM001 or pMUM002 is an inactive DH paired with an AT-III domain. As the DH and KR domains are functionally redundant within the LM, this does not indicate a functional constraint imposed on this pairing, but does suggest that the AT-III and DH domains 'move' as a group. This may also be the case for the ER and KR-A domains that always appear in modules with the same domain organization. This is further highlighted by an intra-species comparison of the domain structure of the load modules of MlsA and MlsB. Individual domains of both the MlsA and MlsB load modules of pMUM002 are identical, with the exception of the AT-III and active DH domains (Figure 7). The simplest explanation for the presence of these domains in otherwise identical sequences is introduction via recombination with the same domain cluster from a neighbouring donor module. Due to the high degree of sequence homology between like domains, any of eight potential modules may have been the donor for the introduced AT-III/DH domain pair (see Figure 7).

**Figure 8:**
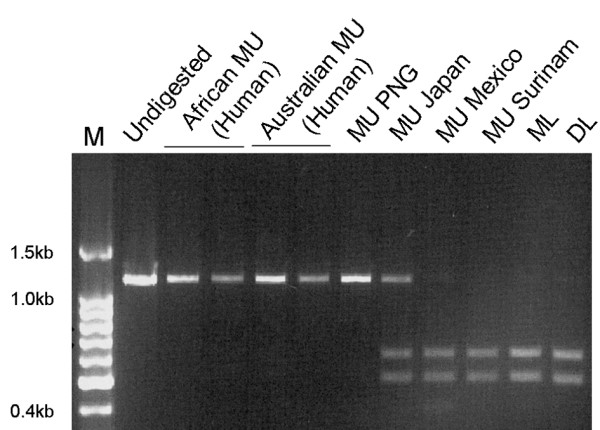
**Ketosynthase domain PCRs from a panel of MU strains**. KS domain sequences were amplified by PCR and the products were digested with *Hin*dIII and separated on 1.2% agarose gel.

To gauge the extent of *mls *sequence homology among different mycolactone-producing mycobacteria (MPM) we took advantage of a single nucleotide polymorphism (SNP) identified within the KS domain during sequencing of pMUM002. Sequencing of the *mls *locus of pMUM002 revealed a *Hin*dIII restriction site in the KS domain of all 15 modules that comprise *mlsA1*, *mlsA2 *and *mlsB*. These *Hin*dIII sites are at the same position within every KS domain and are introduced by a synonymous C → T transition. Curiously, this polymorphism is not present in *any *of the 16 KS domains of pMUM001. The presence of this site within the KS domains of the mycolactone E PKS represented a convenient tool for screening other MPM. Oligonucleotides were designed to the 5' and 3' ends of the KS domain and PCR was used to amplify all KS domains present in a strain. Subsequent restriction digestion with *Hin*dIII of the PCR product obtained from each MPM revealed the presence of this polymorphism in all the KS domains of the South American human MU strains and animal (fish or frog) MPM, with the same profile as ML. However this variation was absent from all the KS domains of African and Australian MU strains, and present in only a proportion of the KS domains from the Japanese strain MU753 (Figure 8). One conclusion from these data is that ongoing intra-strain domain replacement (gene conversion) is purifying the *mls *sequences and resulting in the unusual pan-locus nucleotide homogeneity.

## Discussion

We present here the first detailed description of two recently discovered megaplasmids, harboured by mycolactone-producing mycobacteria isolated from frogs and fish, named pMUM002 (190 kbp) and pMUM003 (~210 kbp) respectively. Sequence analysis revealed that both replicons are highly related to pMUM001 from MU Agy99, with the same overall architecture and at least half their coding potential spanned by the three, large *mls *genes that are required for mycolactone synthesis (Figure 2, Table 1).

Uncovering the genetic basis for the synthesis of mycolactone structural variants by MPM was a major objective of this study. The complete sequence of pMUM002 permitted a thorough analysis of its *mls *locus and an examination of the concordance between module arrangement and the structure of mycolactone E. There was perfect correspondence between the domain and module arrangement for MlsB in pMUM002 and our earlier LC-MS/MS structural predictions that included the presence of a propionate starter unit and only six extender modules (Figure 3). Further support for this structure was also obtained by LC-MS/MS analysis of lipid extracts from two other ML strains and observing the same ion trace and MS/MS fragmentation pattern. Similarly, analysis of a BAC clone that spanned *mlsB *from pMUM003 confirmed mycolactone F structure-based predictions on module and domain composition that included an AT-II (acetate) domain within the load module and the absence of any A-type KR domains; the latter observation explaining the switched stereochemistry of the C11' and C13' side-chain hydroxyls of mycolactone F [5]. Furthermore, the combination of sequencing and LC-MS/MS analysis has shown conclusively that geographically and genetically close MU strains from Japan and China produce different mycolactones (Figure 6). Alternative mycolactone production in all of these strains is due to the highly mutable nature of the mycolactone PKS locus, facilitated by the high levels of nucleotide identity amongst domains, and presumably supporting homologous recombination.

Despite the very high inter-strain nucleotide identity, the *mls *sequences from pMUM001 and pMUM002 form distinct phylogenetic clusters (Figure 7), a separation that correlates with genetic comparisons based on chromosomal genes sequences that show MPM fall into two distinct lineages; the so-called ancestral lineage that includes MPM predominantly infecting fish and frogs and the modern lineage that includes most of the MPM that infect humans [10]. The discrete strain-dependent clustering of the pMUM001 and pMUM002 *mls *domains also suggests that intra- and not inter-strain exchange of homologous domain sequences is the mechanism that has generated metabolite diversity. In fact, the most parsimonious explanation for the *mls *domain and module clustering patterns and the distribution of the *Hin*dIII polymorphism amongst MPM is the evolution of domains or modules in concert and mutations occurring in repeated domains that then become fixed throughout the genes due to gene conversion events [25]. It was originally suggested that the evolution of the multimodular structure of the PKSs is due to repeated rounds of gene duplication from a single ancestor module [26], a notion that has been supported by phylogenetic analysis of KS domains amongst streptomycetes and other PKS pathways [15,27].

Intragenic and intergenic recombination appears to have generated diversity within other PKS clusters, such as the microcystin PKS gene cluster of *Microcystis *sp. [28] and the avermectin and rapamycin clusters of *Streptomyces *sp. [29].

A major stumbling block for combinatorial polyketide biochemistry for production of non-natural products has been the apparent incompatibility of certain domain combinations [30]. In this study we have shown that there is considerable natural tolerance among the Mls domains for a variety of polyketide precursors. For example, by studying the genetic basis for naturally occurring mycolactone variants we have revealed that different AT domains within starter units specify either acetate or propionate, addition or deletion of modules accounts for altered chain length, and the stereochemistry of hydroxyl groups results from a replacement of KR domains, all in accord with the modular PKS paradigm [13]. Furthermore, the high identity among KS domains suggests that they must accept different extender units and many varieties of growing polyketide chain as substrates. In agreement with this, a close comparison of *mls *sequences between pMUM001 & pMUM002 shows that the subtle alterations in KS sequence do not correlate with the type of substrate. Also, domains or modules that perform the same synthesis reactions in different strains are not necessarily the most closely related, again suggesting the KS domains are permissive. For example, the KS domains of MlsA that accept the same mycolactone core substrates in MU Agy99 and ML are not most closely related to each other, as might be predicted if even small changes in the KS alter function, but are more closely related to other KS domains from that strain, which accept different substrates (Figure 7). Exceptions to this are the KS domains from modules 4 and 8 of MlsA of pMUM002, which are most closely related to their counterparts in pMUM001. It is also noteworthy that domain swapping occurs in pairs. The reasons for this are unclear, but one possible explanation is that a pair of adjacent domains provides more favourable conditions for homologous recombination than a single domain.

## Conclusion

In this study DNA sequencing and comparative analysis of pMUM megaplasmids have been used to document the genetic differences in the toxin-coding DNA, and sophisticated mass spectrometry has been used to assign the differences in their chemical structure. The results confirm predictions, and show that highly specific changes in the modular polyketide synthase genes, akin to strategies used in the laboratory to engineer production of altered polyketide antibiotics, account for the differences. Given their uniquely repetitive structure, the genes for these assembly-line multienzymes appear to represent a natural chemistry set that might be harnessed in different modular combinations to create novel polyketides as potential drug leads. Comparative analysis of the repetitive gene structure has also provided clues to the evolutionary events, particularly recombination and gene conversion, that continue to shape these remarkable systems.

## Methods

### Bacterial strains and culture conditions

*M. liflandii *strains 128FXT (ML), XL5 and HW1 were isolated from infected tropical clawed frogs (*Xenopus tropicalis*) at the University of California, Berkeley [31]. *M. marinum *DL240490 (DL) was isolated from a European Sea Bass (*Dicentrarchus labrax*) from the Red sea [32]. MU753 Japan was isolated in 2004 from a diagnosed case of Buruli ulcer from a 37-year old Japanese female [33]. All mycobacterial strains were cultivated by using Middlebrook 7H9 broth or 7H10 agar (Difco) supplemented with oleic acid-albumin-dextrose-catalase (Difco) at 30°C. *E. coli *DH10B (Invitrogen) was cultivated using Luria Bertani broth or agar at 37°C.

### Plasmid cloning, shotgun library construction and sequencing

Prior to sequencing it was anticipated that plasmids from ML and DL would be highly similar in structure to the previously sequenced MU plasmid, pMUM001. So as to avoid confusion with future pMUM-like plasmid sequences and to reflect their common origin, we therefore propose to continue the pMUM nomenclature for all future sequenced mycolactone plasmids. Hence, we have designated the plasmids in this study pMUM002 (from ML) and pMUM003 (from DL). Two bacterial artificial chromosome (BAC) libraries were prepared using the vector pIndigo-BAC5 (Epicentre) for ML and DL as described previously [11]. The resulting *E. coli *DH10B chloramphenicol resistant clones were stored at -80°C in 96-well format in Luria-Bertani broth containing 15% glycerol. To identify BAC clones spanning pMUM DNA, the ML and DL libraries were screened by PCR for two genes found distal to each other on pMUM001, *repA *and MUP038 [11]. BAC clones PCR positive for either gene were then further analysed by end-sequencing and restriction enzyme digestion to construct an overlapping BAC scaffold of each plasmid from ML and DL. Four BAC clones (06A07: 77 kb, 06D10: 110 kb, 07A09: 90 kb, 10A03: 15 kb) spanned all of pMUM002 and three BAC clones spanned all of pMUM003 (04D12: 110 kb, 051B: 99 kb, 048F: 98 kb) (Figure 2). DNA from the BAC clones 06A07, 06D10 and 07A09 were sheared by hydrodynamic shearing (Genomic Solutions Hydroshear), size fractionated to 5 – 7 kb and cloned into the vector pSMART HC Kan (Lucigen Corporation). The selected size of 5 – 7 kb overcome the egregiously repetitive nature of the locus and allowed for the cloning of single PKS modules, the minimum non-repetitive PKS unit. Each subclone was end-sequenced and subclones that represented a single PKS module were subjected to complete sequencing by primer walking. Due to its comparatively small size, BAC clone 10A03 was completely sequenced by primer walking. Unlike BACs which overlapped the PKS region of pMUM002, BAC clone 0412D contained a very limited amount of PKS-encoding DNA and subcloning of this BAC was performed by hydrodynamic shearing followed by a random shotgun approach to clone 2 – 4 kb DNA fragments into pSMART HC kan. Subclones of 0412D were then end-sequenced.

### Bioinformatic analyses

Sequences were assembled using Phrap and Gap4 [34]. Annotation of the nucleotide sequences of pMUM002 and a 110 kb non-PKS region of pMUM003 (from 0412D) were performed as described previously using an in-house web-based database system for genome annotation [12]. The nucleotide sequence of pMUM002 and the sequence of the non-PKS region of pMUM003 have been submitted to GenBank database as EU271968 and EU271967, respectively. Phylogenetic analysis was performed with MEGA 4 software [35]. Dot plots were generated using Dotter [36].

### DNA methods

Methods for PCR, pulsed-field gel electrophoresis and Southern hybridisation were performed as described previously [37]. Southern hybridisation probes for both type A and B ketoreductase domains (KR-A and -B, respectively) of the mycolactone PKS were based on regions of divergent sequence within each enzymatic domain. The KR-A probe (aaggtggttggcccacaaatatgaatcggtag) recognised the nucleotide sequence specifying RWLAHKYESV, whilst the KR-B probe (cgagcatctggtttctgcccatggtgtccggc) recognised the nucleotide sequence specifying EHLVSAHGVR. Oligonucleotide probes were labelled using the DIG oligonucleotide tailing kit (Roche). BAC clones A04 and D03 from pMUM001 (Figure 2) have previously been published [17].

### Lipid extraction and analysis

Lipid fractions of ML were extracted and analysed for mycolactones as previously described using a Finnigan LCQ (Thermo Finnigan, USA) ion-trap mass spectrometer, coupled with a HP1100 liquid chromatography. Mycolactones were eluted from a ThermoHypersil BDS C8 column (5 μm, 4.6 × 250 mm) with a gradient of 55 to 95% acetonitrile in water over 40 min [38,39].

## Abbreviations

PKS: polyketide synthase; CDS: Protein-coding DNA sequence; BAC: bacterial artificial chromosome.

## Authors' contributions

SJP carried out DNA sequencing, sequence assembly, sequence analysis, other molecular genetic analyses and co-wrote the manuscript. HH conducted mycolactone structural analyses. TS performed bioinformatic analysis. JLP, MJY, AM, MJ, and PW assisted with DNA sequencing. JKD and PFL participated in study design and data analysis. TPS conceived the study and conducted DNA sequence assembly, sequence analysis and co-wrote the manuscript. All authors read and approved the final manuscript.

## Supplementary Material

Additional file 1 **Shared DNA between pMUM plasmids (A) Dot plots showing a two-way comparison of DNA identity over the length of the non-PKS region of pMUM001, pMUM002 and pMUM003.** (B) Venn diagram showing the shared and unique CDS amongst the three pMUM plasmids. Click here for file

Additional file 2 **Description of other features of pMUM001 and pMUM002, not directly associated with mycolactone synthesis.** Click here for file

Additional file 2 **Table summary of the 96 predicted CDS in pMUM002.** Click here for file

Additional file 4 **ClustalW alignment**. Alignment of the derived amino acid sequences for the AT domains from the load modules of MlsA1 and MlsB from *M. liflandii *128FXT, showing the AT domain from MlsB has a sequence consistent with methylmalonate specificity. Boxed sequences are residues known to be critical for AT substrate specificity. Click here for file
