# Supplementary figures and images for: Deciphering the genetic basis for polyketide variation among mycobacteria producing mycolactones
Source: BMC Genomics. 2008 Oct 7;9:462. doi: 10.1186/1471-2164-9-462 (PMC2569948; doi:10.1186/1471-2164-9-462)

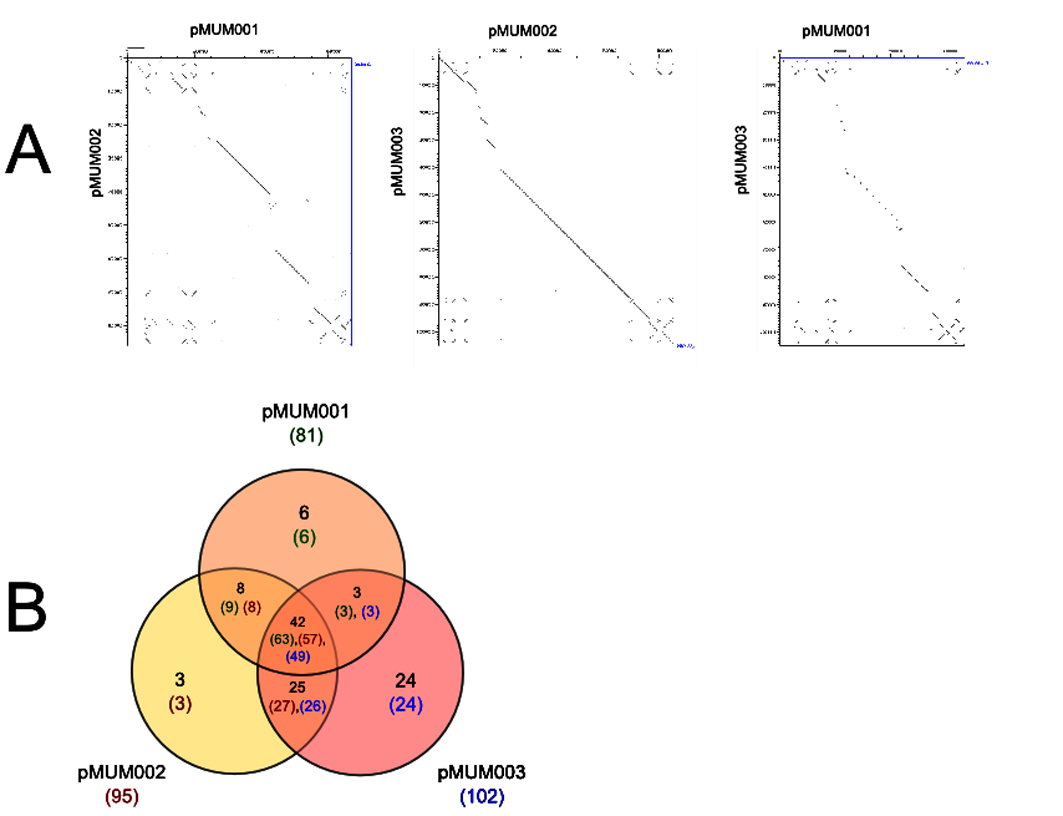

Supplement: Additional file 1 — Shared DNA between pMUM plasmids (A) Dot plots showing a two-way comparison of DNA identity over the length of the non-PKS region of pMUM001, pMUM002 and pMUM003. (B) Venn diagram showing the shared and unique CDS amongst the three pMUM plasmids. [file 1471-2164-9-462-S1.tiff]

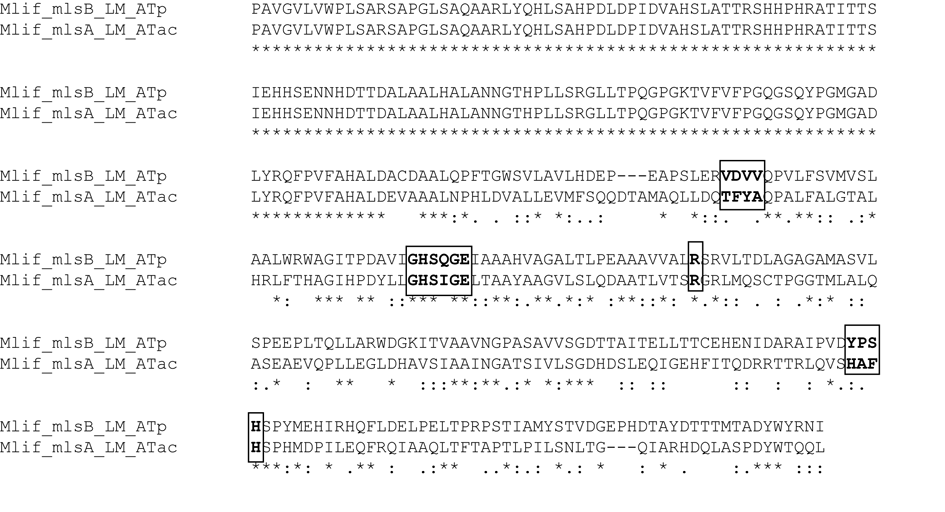

Supplement: Additional file 4 — ClustalW alignment. Alignment of the derived amino acid sequences for the AT domains from the load modules of MlsA1 and MlsB from M. liflandii 128FXT, showing the AT domain from MlsB has a sequence consistent with methylmalonate specificity. Boxed sequences are residues known to be critical for AT substrate specificity. [file 1471-2164-9-462-S4.tiff]
