# Supplementary material for: Deciphering the genetic basis for polyketide variation among mycobacteria producing mycolactones
Source: BMC Genomics. 2008 Oct 7;9:462. doi: 10.1186/1471-2164-9-462 (PMC2569948; doi:10.1186/1471-2164-9-462)
Supplement: Additional file 2 — Description of other features of pMUM001 and pMUM002, not directly associated with mycolactone synthesis. [file 1471-2164-9-462-S2.rtf]

Additional text describing some of the pMUM002 and pMUM003 CDS not known to be associated with mycolactone biosynthesis

General descriptions of pMUM002 and pMUM003
Comparisons with pMUM001 suggest a mosaic structure for pMUM replicons with regions of extensive conservation surrounding areas of critical function such as the origin of replication and the mls loci. There is a high degree of synteny surrounding the replication region among all the pMUM plasmids, with a large stretch of DNA extending 6kb upstream and 10kb downstream of repA, conserved in both gene content and order. However, there are also some regions of difference. For instance, pMUM002 and pMUM003 share an additional 25 CDS that are not found on pMUM001 (Supplementary Figure 1). These 25 CDS are found scattered throughout the plasmid DNA and range in size from 0.15kb to 15.0kb (Supplementary Figure 1).

The megaplasmid pMUM002 also contains 28 copies of insertion sequence elements (ISE) or fragments of ISE, many of which have been previously identified in pMUM001. Twelve CDS were predicted to be membrane associated proteins, seven of which have orthologs in pMUM001. There were 46 CDS annotated as hypothetical proteins, of which 39 have been assigned as conserved hypothetical proteins due to their homology with hypothetical proteins in M. ulcerans (32), Mycobacterium tuberculosis (2), Mycobacterium vanbaalenii PYR-1 (1), Mycobacterium flavescens PYR-GCK (1), Mycobacterium gilvum (1), Mycobacterium sp. JLS (1), and Mycobacterium smegmatis (1).  The remaining seven hypothetical proteins have no homology to any sequences in the public databases. 

The megaplasmid pMUM003 contains at least 18 copies of ISE or fragments of ISE, all of which are homologous to those found on pMUM001 or pMUM002. Eight CDS were predicted to encode membrane associated proteins, four of which have orthologs in either pMUM001 or pMUM002. There were 65 CDS annotated as hypothetical proteins, of which 45 have orthologs in pMUM001 or pMUM002. Of the remaining 20 hypothetical proteins, 13 had orthologs in other actinobacteria, whilst seven had no homology to any sequences in the public databases. 

Each of pMUM001, pMUM002 and pMUM003 possess genes encoding one or more putative serine/threonine signal transduction protein kinases (STPKs), proteins that have been found to regulate cellular responses to environmental signals in diverse bacterial genera [1]. One of these putative STPKs is present on all three plasmids (MUP011, MULP_022, MUDP_075), although a frame-shift mutation suggests MUDP_075 is a pseudogene. Both pMUM002 and pMUM003 share another STPK (MULP_016, MUDP_069), whilst pMUM003 also contains a third putative STPK (MUDP_015). These proteins show high levels of homology to other mycobacterial STPKs in the N-terminus, however, there are no matches to the C-terminal sequences in public databases, suggesting that they each act on unique substrates. 

Several mycobacterial STPK genes (pkn) have been studied in detail and some have been shown to be essential for the growth of M. tuberculosis [2-4]. Furthermore, other mycobacterial STPKs have been shown to phosphorylate proteins containing forkhead-associated (FHA) domains [5-9]. FHA domains contain phosphothreonine-binding motifs and whilst their functions have been characterised in cellular processes such as signalling DNA damage, vesicular transport, and cell cycle control in eukaryotes, their roles in prokaryotes are much less well defined [1, 10-11]. All of the pMUM plasmids examined to date contain one putative FHA domain protein (MUP018, MULP_029 and MUDP_081), although no experimental data is available to show an association with any of the STPKs found on the plasmids. The environmental signals that may be recognised by these signal transducers remain to be discovered but the conservation of these potential regulatory loci suggest that they may be playing an essential role, perhaps in someway linked to mycolactone synthesis.

No functional plasmid transfer loci were identified on either pMUM002 or pMUM003. However, pMUM003 does possess an FtsK/SpoIIIE domain protein (MUDP_038) that, due to a frame-shift mutation, has become a pseudogene. MUDP_038 comprises part of an 8.4kb region that has been deleted from both pMUM001 and pMUM002. In Streptomyces spp. the plasmid-encoded FtsK/SpoIIIE domain protein, TraB, is involved in plasmid transfer [12-14].  MUDP_038 has similarity to FtsK/SpoIIIE domain proteins from other actinobacteria and it is possible that this gene may have formed part of an ancestral but now defunct pMUM transfer system. PCR screening and sequence analysis of MUDP_038 between different MPM has shown that in those strains that harbour this CDS, it is also probably a pseudogene. 


References

[1] Greenstein AE, Grundner C, Echols N, Gay LM, Lombana TN, Miecskowski CA, Pullen KE, Sung PY, Alber T (2005) Structure/function studies of Ser/Thr and Tyr protein phosphorylation in Mycobacterium tuberculosis. J Mol Microbiol Biotechnol 9:167-181

[2] Sassetti CM, Boyd DH, Rubin EJ (2003) Genes required for mycobacterial growth defined by high density mutagenesis. Mol Microbiol 48:77-84

[3] Walburger A, Koul A, Ferrari G, Nguyen L, Prescianotto-Baschong C, Huygen K, Klebl B, Thompson C, Bacher G, Pieters J (2004) Protein kinase G from pathogenic mycobacteria promotes survival within macrophages. Science 304:1800-1804

[4] Kang CM, Abbott DW, Park ST, Dascher CC, Cantley LC, Husson RN (2005) The Mycobacterium tuberculosis serine/threonine kinases PknA and PknB: substrate identification and regulation of cell shape. Genes Dev 19:1692-1704

[5] Umeyama T, Lee PC, Horinouchi S (2002) Protein serine/threonine kinases in signal transduction for secondary metabolism and morphogenesis in Streptomyces. Appl Microbiol Biotechnol 59:419-425

[6]    Molle V, Kremer L, Girard-Blanc C, Besra GS, Cozzone AJ, Prost JF (2003) An FHA phosphoprotein recognition domain mediates protein EmbR phosphorylation by PknH, a Ser/Thr protein kinase from Mycobacterium tuberculosis. Biochemistry 42:15300-15309


[7]   Curry JM, Whalan R, Hunt DM, Gohil K, Strom M, Rickman L, Colston MJ, Smerdon SJ, Buxton RS (2005) An ABC transporter containing a forkhead-associated domain interacts with a serine-threonine protein kinase and is required for growth of Mycobacterium tuberculosis in mice. Infect Immun 73:4471-4477


[8]   Grundner C, Gay LM, Alber T (2005) Mycobacterium tuberculosis serine/threonine kinases PknB, PknD, PknE, and PknF phosphorylate multiple FHA domains. Protein Sci 14:1918-1921

[9]   Villarino A, Duran R, Wehenkel A, Fernandez P, England P, Brodin P, Cole ST, Zimny-Arndt U, Jungblut PR, Cervenansky C, Alzari PM (2005) Proteomic identification of M. tuberculosis protein kinase substrates: PknB recruits GarA, a FHA domain-containing protein, through activation loop-mediated interactions. J Mol Biol 350:953-963

[10]  Durocher D, Jackson SP (2002) The FHA domain. FEBS Lett 513:58-66

[11]  Hammet A, Pike BL, McNees CJ, Conlan LA, Tenis N, Heierhorst J (2003) FHA domains as phospho-threonine binding modules in cell signaling. IUBMB Life 55:23-27

[12]  Pettis GS, Cohen SN (1996) Plasmid transfer and expression of the transfer (tra) gene product of plasmid pIJ101 are temporally regulated during the Streptomyces lividans life cycle. Mol Microbiol 19:1127-1135

[13]  Grohmann E, Muth G, Espinosa M (2003) Conjugative plasmid transfer in gram-positive bacteria. Microbiol Mol Biol Rev 67:277-301

[14]  Reuther J, Wohlleben W, Muth G (2006) Modular architecture of the conjugative plasmid pSVH1 from Streptomyces venezuelae. Plasmid 55:201-20
