# Supplementary material for: Deciphering the genetic basis for polyketide variation among mycobacteria producing mycolactones
Source: BMC Genomics. 2008 Oct 7;9:462. doi: 10.1186/1471-2164-9-462 (PMC2569948; doi:10.1186/1471-2164-9-462)
Supplement: Additional file 2 — Table summary of the 96 predicted CDS in pMUM002. [file 1471-2164-9-462-S3.rtf]

Additional file 3: Table summary of the 96 predicted CDS in pMUM002
CDS	Coordinates	Sense	Start codon	G+C (%)	Predicted protein size (aa)	Predicted product	Closest ortholog	aa identity in overlap (%), size of overlap (aa)	Protein domains / families	
MULP_001 repA	1 - 1239	+	M	66.2	412	Replication protein	RepA (MUP001) 	98, 361		
MULP_002	1109 – 1423	-	M	62.5	104	Conserved hypothetical protein	MUP002	100, 104		
MULP_003	1686 – 2282	+	V	62.1	198	Conserved hypothetical protein	MUP003	98, 198	One HTH domain	
MULP_004	2302 – 2916	-	V	62.0	204	Conserved hypothetical protein	MUP004	98, 204		
MULP_005 parA	2913 – 3893	-	M	57.8	326	Chromosome partitioning protein	ParA (MUP005)	99, 326	ParA family
ATPase	
MULP_006	5320 – 6387	+	V	66.1	355	P60 related protein	Mycobacterium sp. KMS NLP/P60	62, 112	NLPc/P60 family
Cell-wall associated hydrolase motif	
MULP_007	6338 – 6982	-	V	65.3	214	Hypothetical protein	Hypothetical alanine rich protein M. flavescens PYR-GCK	37, 87		
MULP_008	7329- 7739	+	M	63.5	136	Transcriptional regulatory protein	MUP021	100, 87	One HTH domain	
MULP_009	7732 – 8244	+	V	66.7	170	Hypothetical protein				
MULP_010	8241 – 8780	+	V	60.6	179	Hypothetical protein				
MULP_011	8777 – 9331	+	M	64.3	184	Nucleic acid binding protein	MUP008	97, 184	NusA family	
MULP_012	9557 – 10237	+	M	62.4	226	Conserved  hypothetical protein	MUP006	99, 212		
MULP_013	10374 – 15299	+	M	63.4	1641	DNA methylase	putative helicase; putative methylase Gordonia westfalica	45, 1400	DNA methylase
Contains one HTH domain	
MULP_014	15145 – 15747	+	M	63.2	200	Hypothetical protein			Contains two HTH domains	
MULP_015	16300 – 17013	+	M	63.0	237	Hypothetical protein	hypothetical protein Mflv_5469 M. gilvum	42, 129		
MULP_016	17284 – 18993	+	M	67.5	569	Serine/Threonine protein kinase	M. tuberculosis Haarlem pknJ	41, 577	Ser/Thr kinase catalytic domain One TMD	
MULP_017	19017 – 19496	+	M	62.5	159	Hypothetical membrane protein	M. ulcerans MUL_3582	30, 80		
MULP_018	19569 – 20462	-	V	61.1	298	Hypothetical protein				
MULP_019	20613 – 20837	+	V	66.7	74	Transcriptional regulator	Frankia spp. CcII3 marR	38, 74		
MULP_020	21205 – 22380	+	M	66.8	391	Hypothetical protein	Mycobacterium sp. JLS MJLS_Draft0294	30, 396		
MULP_021	22377 – 23600	+	M	65.6	407	Hypothetical protein	MUP009; MUP010	87, 93; 98, 263	Probable signal seq	
MULP_022	23633 – 25588	+	V	64.8	652	Serine/Threonine protein kinase	MUP011	98, 652	One TMD	
MULP_023	25442 – 26323	-	M	60.1	293	Hypothetical membrane protein	MUP012; MUP013	98, 81; 100, 90	Probable signal seq, three TMDs	
MULP_024	26325 – 27098	-	V	66.1	257	Integral membrane protein	MUP014	98, 257	Five TMDs	
MULP_025	26803 – 27870	-	M	65.9	355	Secreted protein	MUP015	99, 252	Probable signal seq	
MULP_027	27867 – 28658	-	V	65.7	263	Conserved hypothetical protein	MUP016	99, 263	Probable signal seq	
MULP_028	28655 – 29113	-	M	64.5	153	Membrane protein	MUP017	98, 152	One TMD	
MULP_029	29157 – 30245	-	M	65.0	362	Forkhead-associated protein	MUP018	99, 362	FHA domain	
MULP_030	30590 – 31624	+	M	63.5	344	Conserved membrane protein	MUP019	98, 344	One TMD	
MULP_031	31614 – 31796	+	M	58.5	60	Conserved hypothetical protein	MUP020	96, 60		
MULP_032	31793 – 32203	+	M	63.5	136	Transcriptional regulatory protein	MUP021	100, 87	One HTH domain	
MULP_033	32196 – 32645	+	V	60.0	149	Hypothetical protein	MUP020	54, 37	Three TMDs	
MULP_034	32783 – 33112	+	M	59.4	109	Hypothetical protein			Two TMDs	
MULP_035	33718 – 34620	-	M	61.8	300	IS1547-like transposase, pseudogene 	M. avium subsp. paratuberculosis K-10 IS1547_2 (367)	65, 367	Transposase family
	
MULP_036	34586 – 35633	-	M			IS2404 (fragments)			Pseudogene	
										
MULP_038	36238 - 36771	-	M	59.9	177	Hypothetical protein	M. vanbaalenii Mvan_3731	36, 167		
MULP_039	36998 - 37207	-	M	60.0	69	Hypothetical protein				
MULP_040	37475 - 37900	-	M	65.0	141	Hypothetical protein				
MULP_041	38481 - 38741	+	M	63.6	86	Hypothetical protein			One TMD	
MULP_042	39694 - 40326	-	V	64.8	210	Conserved hypothetical protein	MUP023	99, 210		
MULP_043	40323 40910	-	M	64.8	195	Conserved hypothetical protein	MUP024	98, 195		
MULP_044	41452 - 43149	+	L	63.8	565	Transposase	MUP025 / MUP028	99, 521	Transposase family	
MULP_045	43232 - 44566	+	M	61.9	444	IS2606	IS2606 M. ulcerans	99, 444		
MULP_046	44756 – 45803	-	M			IS2404 (fragments)			Pseudogene	
MULP_047	46142 - 46960	-	V	64.5	272	Transposase	MUP027	97, 272	IS116/IS110/IS902 family	
MULP_048	47088 - 48833	-	V	63.7	581	Transposase	MUP025 / MUP028	99, 521		
MULP_049	49137 – 50183	+	M	61.8	348	IS2404b	IS2404	97, 348	Contains in-frame stop	
MULP_050	50432 - 50791	-	V	66.4	119	Hypothetical protein	M. tuberculosis F11 hypothetical protein	81, 54		
MULP_051 mlsB	50788 - 87846	-	V	62.6	12352	Type I polyketide synthase				
MULP_052	87972 - 88352	-	V	62.7	124	Transposase	MUP033 / MUP041	95, 124	Transposase family	
MULP_053	88450 – 88928	-	M	61.3	160	Transposase	MUP034 / MUP042	99, 125	Pseudogene	
MULP_054	88950 – 89997	-	M			IS2404 (fragments)			Pseudogene	
										
MULP_056	90273 - 90359	-	M	52.9	28	Transposase fragment	MUP034 / MUP042	95, 22	Pseudogene	
MULP_057	90547 - 90687	-	M	67.4	46	Transposase fragment	MUP052	65, 46	Pseudogene	
MULP_058	91001 - 92056	+	M	66.5	351	Transposase	MUP035 / MUP043	98, 351		
MULP_059	92046 - 92186	-	M	62.4	46	Transposase fragment	MUP044	100, 46	Pseudogene	
MULP_060	92560 - 94305	-	V	63.7	581	Transposase	MUP025 / MUP028	99, 545		
MULP_061	94354 - 95400	-	M	61.8	348	Transposase	IS2404	97, 348		
										
MULP_063	96291 - 97196	-	M	64.2	301	Type II thioesterase	MUP038	99, 301		
MULP_064 mlsA2	97352 - 104584	-	V	62.0	2410	Type I polyketide synthase			One HTH domain	
MULP_065 mlsA1	104571 - 155630	-	V	63.2	17019	Type I polyketide synthase				
MULP_066	155722 - 156096	-	V	62.7	124	Transposase	MUP033, MUP041	95, 124	Transposase family	
MULP_067	156194 – 156732	-	V			Transposase (fragments)	MUP034 / MUP042	98, 145	Pseudogene	
MULP_068	156914 - 157054	-	L	67.4	46	Transposase, fragment	MUP052	65, 46	Pseudogene	
MULP_069	157149 – 157972	+	M			IS2404 (fragments)	IS2404		Pseudogene	
MULP_070	158225 - 159211	+	V	52.8	328	Type III keto-synthase	MUP045	99, 328	FabH domain	
MULP_071	159099 - 159650	-	M	59.2	183	C-term transposase	MUP082	99, 183	Pseudogene	
MULP_072	159839 – 160348	+	V	56.9	67	IS2606 fragment	IS2606		Pseudogene	
MULP_073	160314 – 161361	-	M			IS2404 (fragments)	IS2404		Pseudogene	
MULP_076	162038 - 163156	-	V	62.9	372	Lipoprotein	MUP057	98, 258	Probable signal seq	
MULP_077	163353 - 164390	-	V	60.2	345	Site-specific recombinase	MUP058	99, 345	XerC/D integrase family	
MULP_078	164837 - 165884	+	M			IS2404 (fragments)			Pseudogene	
MULP_079	165910 - 166905	+	V	63.4	331	ERCC4 domain protein	ERCC4 domain protein M. gilvum PYR-GCK	73, 328	ERCC4-type nuclease domain	
MULP_080	166958 - 167743	-	M	62.5	261	IS sequence	Rhodococcus erythropolis IS sequence	64, 256	DnaC replication protein domain	
MULP_081	167740 - 169014	-	V	64.1	424	C-term transposase	putative transposase R. erythropolis		Pseudogene	
MULP_082	169490 - 170182	+	V	62.0	230	N-term transposase	putative transposase R. erythropolis		Pseudogene	
MULP_083	170223 - 171848	-	M	62.9	541	Conserved hypothetical protein	MUP065	100, 229	Possible pseudogene
Helicase domain	
MULP_084	172389 - 172925	-	M	60.3	178	Conserved hypothetical protein	MUP066	98, 168		
MULP_085	173007 - 173639	+	V	69.4	210	ATP-dependent exoDNase	M. vanbaalenii Atp-dependent exoDNase	38, 205	Possible pseudogene
Two HTH segments	
MULP_086	174754 - 176103	-	V	65.7	450	Hypothetical protein	M. smegmatis MSMEG_1517	29, 307	Five HTH segments	
MULP_087	176091 - 176615	-	M	64.8	174	Hypothetical protein				
MULP_088	176754 - 177767	-	V	58.4	337	ATPase	Nocardia farcinica nfa8260	35, 340	Possible pseudogene
One HTH domain	
MULP_089	177976 - 178380	-	M	59.0	134	Hypothetical protein	M. tuberculosis Rv1784	38, 109	Possible pseudogene
One TMD	
MULP_090	178543 - 180042	-	V	64.3	499	Membrane protein	MUP068	92, 351	One TMD	
MULP_091	180219 - 180965	-	V	59.3	248	Conserved hypothetical protein	MUP070	92, 246		
MULP_092	181142 - 181498	-	M	59.4	118	Conserved hypothetical protein	MUP071	98, 112		
MULP_093	181592 - 183979	-	M	66.8	795	Hypothetical protein	MUP072, MUP073	98, 398 ; 96, 354	Two HTH domains	
MULP_094	184001 - 184584	-	M	66.0	197	Conserved hypothetical protein	MUP074		Pseudogene	
MULP_095	184581 - 184919	-	M	66.1	112	Conserved hypothetical protein	MUP075	99, 112		
MULP_096	184919 - 185590	-	M	64.1	223	Conserved hypothetical protein	MUP076	99, 223	One TMD	
MULP_097	185624 - 186016	-	V	59.8	130	Conserved hypothetical protein	MUP077	98, 130		
MULP_098	186013 - 18773	-	V	66.6	586	Conserved hypothetical protein	MUP078	98, 467	Probable signal seq	
MULP_099	187792 - 188091	-	M	62.7	99	Conserved hypothetical protein	MUP079	100, 99		
MULP_100	188098 - 188370	-	M	65.9	90	Conserved hypothetical protein	MUP080	100, 90	One HTH domain	
MULP_101	188444 - 189979	-	M	65.0	511	Conserved hypothetical protein	MUP081	99, 503		


Supplementary Table S2. Summary of the 103 predicted CDS in pMUM003 non-PKS region.
CDS	Coordinates	Sense	Start codon	G+C (%)	Predicted protein size (aa)	Predicted product	Closest ortholog (size)	aa identity in overlap (%), size of overlap (aa)	Protein domains / families	
MUDP_001	132 - 506	-	V	62.9	124	Transposase	MUP041 (124)	100, 124	Transposase family	
MUDP_002	604 – 1136	-	V	59.1		Transposase, pseudogene	MUP034 / MULP_067		Pseudogene	
MUDP_003	1324 – 1593	-	V	61.9	89	Transposase, fragment	MULP_068 / MUP002		Pseudogene	
MUDP_004	1613 – 2089	+	V	61.4	158	C-term IS2404, fragment	IS2404		Pseudogene	
MUDP_005	2397 – 3383	+	V	52.7	328	Type III keto-synthase	MULP_070 (328)	99, 328	FabH domain	
MUDP_006						C-term transposase, fragment			Pseudogene	
MUDP_007	4008 – 4421	+	M	59.4	137	C-term IS2606, fragment	IS2606		Pseudogene	
MUDP_008	4754 – 5800	-	M	61.8	348	IS2404	IS2404 (348)	98, 347	Transposase family	
MUDP_009	6018 – 6452	-	V	62.3	144	Conserved hypothetical protein	MUP056 (126)	98, 120		
MUDP_010	6551 – 7328	-	V	62.9		Possible lipoprotein	MUP057 (258)	93, 186	Pseudogene ??	
MUDP_011	7525 – 8562	-	V	60.2	345	Site specific recombinase	MUP058 / MULP077 (345)	99, 345	XerC/D integrase family	
MUDP_012	8705 – 9760	+	V	63.6	321	ERCC4 domain protein	MULP_079 (331)	99, 321	ERCC4 domain	
MUDP_013	9753 – 10538	-	M	62.6	261	IS sequence	MULP_080 (261)	99, 261	DnaC replication protein domain	
MUDP_014	10535 - 12106 	-	V	63.9	523	Transposase	putative transposase R. erythropolis (559)	55, 540	Transposase family	
MUDP_015	12826 – 14700	+	M	60.7	624	Serine/Threonine protein kinase	PknF M. ulcerans (495)	60, 329		
MUDP_016	15031 – 15477	-	V	63.5	148	Hypothetical protein	M. tuberculosis Haarlem lpqH (159)	42, 152	Predicted signal seq	
MUDP_017	15765 – 16049	+	V	59.3	94	Hypothetical protein	M. avium excalibur (60)	63, 38	One TMD	
MUDP_018	16076 – 16948	+	M	61.3	290	Hypothetical secreted protein 	Rhodococcus sp. RHA1 hypothetical protein (360)	62, 248	Predicted signal seq	
MUDP_019	17146 – 17553	-	V	59.3	135	Conserved membrane protein	MUP064 (120)	98, 115	Three TMDs	
MUDP_020	17764 – 18240	+	L	58.5	158	N-term transposase	MULP_082 (230)	100, 93	Pseudogene	
MUDP_021	18265 – 18606	-	L	60.2	113	Conserved hypothetical protein	MULP_083 (541)	98, 106	Pseudogene	
MUDP_022	18648 – 19055	-	M	57.4	135	Hypothetical protein	M. smegamatis MSMEG_1724 (135)	54, 122		
MUDP_023	19152 – 19781	-	V	61.1	209	Glycosylase	Mycobacterium sp. MCS MMCS_0026 (222)	52, 199	Uracil-DNA glycosylase family	
MUDP_024	19866 – 20213	-	V	61.5	115	Hypothetical protein	Rhodococcus sp. RHA1 RHA1_ro05939 (125)	34, 119		
MUDP_025	20490 – 23066	-	M	64.1	858	Conserved hypothetical protein	MULP_083 (541)	99, 427	DinG/Rad3 helicase family	
MUDP_026	23607 - 24113	-	V	60.6	169	Conserved hypothetical protein	MUP066 (168)	99, 168		
MUDP_027	24200 - 24607	-	M	68.1	135	Hypothetical protein	M. gilvum Mflv_2793 (191)	39, 123		
MUDP_028	24961 - 30823	+	M	63.8		ATP-dependent exoDNase, fragments	M. vanbaalenii PYR-1 (1952)		Pseudogene	
MUDP_029	31179 - 31544	+	M	60.4	121	Hypothetical protein				
MUDP_030	32703 - 31670	-	M	66.4		Conserved hypothetical protein, pseudogene	MULP_086 (449)	96, 166	Pseudogene	
MUDP_031	33015 - 33959	-	V	66.2	314	Hypothetical protein	M. avium MAP1509 (300)	26, 318		
MUDP_032	33956 - 34240	-	M	61.4	94	Hypothetical protein	M. leprae (ML1180) (95)	29, 62		
MUDP_033	34291 - 34593	-	M	61.4	101	Hypothetical protein	Mycobacterium sp. KMS MKMS_5775 (106)	32, 91		
MUDP_034	34672 - 34941	-	V	70.4	89	Hypothetical protein				
MUDP_035	35141 - 35971	-	M	68.5	276	Hypothetical protein	M. marinum MMAR_4501 (390)	36, 275	Possible PPE family protein	
MUDP_036	36178 - 36474	-	M	69.0	98	Hypothetical protein	M. avium MAP0157 (103)	40, 87		
MUDP_037	36622 - 37029	-	V	67.2	135	Hypothetical protein	MUP018 (362)	35, 64		
MUDP_038	37026 - 41154	-	V	60.1		FtsK/SpoIIIE family protein, pseudogene				
MUDP_039	41253 – 42170	-	V	64.8	305	Conserved hypothetical protein, pseudogene	MULP_090 (499)	99, 305	Pseudogene	
MUDP_040	42250 – 42714	+	M	62.8	154	Hypothetical protein				
MUDP_041	42929 – 43927	-	V	58.7	332	Conserved hypothetical protein	MULP_091 (248)	99, 248		
MUDP_042	44303 – 46630	-	M	67.7		Conserved hypothetical protein, pseudogene	MULP_093 (795)	96, 621	Pseudogene	
MUDP_043	47244 – 46661	-	M	63.2		Conserved hypothetical protein, pseudogene	MULP_094 (pseudogene)		Pseudogene	
MUDP_044	47241 – 47582	-	V	66.1	113	Conserved hypothetical protein	MULP_095 (113)	100, 112		
MUDP_045	47579 – 48250	-	M	64.3	223	Conserved membrane protein	MULP_096 (223)	99, 199	1 TMD	
MUDP_046	48284 – 48676	-	V	59.8	130	Conserved hypothetical protein	MULP_097 (130)	99, 130		
MUDP_047	49924 - 48673	-	V	64.6		Conserved hypothetical protein, pseudogene	MULP_098 (586)		Pseudogene	
MUDP_048	49929 – 50339	+	M	69.8	136	Hypothetical protein	M. avium MAP4326	29, 144		
MUDP_049	50453 – 50752	-	M	62.3	99	Conserved hypothetical protein	MULP_099 (99)	98, 99		
MUDP_050	50759 – 51031	-	M	65.9	90	Conserved hypothetical protein	MULP_100 (90)	100, 90	One HTH domain	
MUDP_051	51105 – 52640	-	M	64.9	511	Conserved hypothetical protein	MULP_101 (511)	99, 511		
MUDP_052	53250 – 54356	+	M	66.8	368	Replication protein, RepA	MUP001 (368)	98, 368		
MUDP_053	54366 – 54680	-	M	62.5	104	Conserved hypothetical protein	MUP002 (104) / MULP_002 (104)	99, 104		
MUDP_054	54943 – 55539	+	V	62.0	198	Conserved hypothetical protein	MUP003 (198) / MULP_003 (198)	98, 198	One HTH domain	
MUDP_055	55559 – 56173	-	V	61.8	204	Conserved hypothetical protein	MULP_004 (204)	99, 204		
MUDP_056	56170 – 57150	-	M	57.7	326	Partitioning protein, ParA	MULP_005 (326)	100, 326 		
MUDP_057	57940 – 58200	+	V	59.4	86	Hypothetical protein				
MUDP_058	58579 – 59637	+	V	65.9	352	Conserved hypothetical protein	MULP_006 (355)	98, 355	NLPC/P60 domain
Cell-wall associated hydrolase domain	
MUDP_059	59588 – 60232	-	M	65.3	214	Conserved hypothetical protein	MULP_007 (214)	100, 214		
MUDP_060	60579 – 60989	+	M	63.7	136	Putative transcriptional regulatory protein	MULP_008 (136)	98, 136	One HTH domain	
MUDP_061	60982 – 61494	+	V	66.5	170	Conserved hypothetical protein	MULP_009 (170)	99, 170		
MUDP_062	61491 – 62030	+	V	60.7	179	Conserved hypothetical protein	MULP_010 (179)	99, 179		
MUDP_063	62027 – 62575	+	M	63.9	182	Nucleic acid binding protein	MULP_011 (184)	98, 184	NusA family protein	
MUDP_064	62583 – 62804	+	M	64.9	73	Conserved hypothetical protein	MUP007 (73)	98, 73		
MUDP_065	62801 – 63574	+	M	62.4	257	Conserved hypothetical protein	MULP_012 (226)	99, 226		
MUDP_066	63619 – 68991	+	M			DNA methylase, pseudogene			Pseudogene	
MUDP_067	69050 – 69319	+	M	61.9	89	Hypothetical protein				
MUDP_068	69504 – 70256	+	V	63.5	250	Conserved hypothetical protein	MULP_015 (237)	98, 237		
MUDP_069	70464 – 72236	+	M	67.3	590	Ser/Thr protein kinase, PknJ	MULP_016 (569)	99, 569	One TMD	
MUDP_070	72260 – 72739	+	M	62.1	159	Conserved hypothetical protein	MULP_017 (159)	100, 159		
MUDP_071	72812 - 73774	-	M	61.3	320	Conserved hypothetical protein	MULP_018 (297)	99, 296		
MUDP_072	73856 – 74080	+	V	66.7	74	Conserved hypothetical protein	MULP_019 (74)	98, 74		
MUDP_073	54447 – 75622	+	M	66.8	391	Conserved hypothetical protein	MULP_020 (391)	100, 391		
MUDP_074	75619 – 76842	+	M	65.4	407	Conserved hypothetical protein	MULP_021 (407)	99, 407	Predicted signal sequence	
MUDP_075	76875 – 78832	+	M			Ser/Thr protein kinase, pseudogene				
MUDP_076	78849 – 79568	-	M	60.4	239	Conserved membrane protein	MULP_023 (233)	100, 233	Four TMDs
Predicted signal sequence	
MUDP_077	79570 – 80253	-	V	65.9	227	Conserved membrane protein	MULP_024 (257)	100, 230	Four TMDs	
MUDP_078	79947 – 81116	-	M	66.1	389	Conserved secreted protein	MUP015 (258)	98, 252	Predicted signal sequence	
MUDP_079	81113 – 81826	-	V	65.3	237	Conserved hypothetical protein	MULP_027 (263)	99, 238		
MUDP_080	81899 – 82351	-	M	64.5	150	Conserved hypothetical protein	MUP017 (150)	100, 150	One TMD	
MUDP_081	82395 – 83483	-	M	65.1	362	Forkhead-associated protein	MULP_029 (362)	362, 100	FHA Domain	
MUDP_082	83911 – 84855	+	M	63.6	314	Conserved hypothetical protein	MULP_030 (344)	97, 192		
MUDP_083	84845 – 85027	+	M	58.5	60	Conserved hypothetical protein	MULP_031 (60)	100, 60		
MUDP_084	85024 – 85434	+	M	63.7	136	Transcriptional regulatory protein	MULP_032 (136)	99, 136	One HTH	
MUDP_085	85427 – 85876	+	V	59.6	149	Conserved hypothetical protein	MULP_033 (149)	98, 149	Three TMDs	
MUDP_086	86014 – 86343	+	M	59.7	109	Conserved hypothetical protein	MULP_034 (109)	99, 109	Two TMDs	
MUDP_087	86498 – 86692	+	M	61.0	64	Hypothetical protein				
MUDP_088	86943 – 89195	+	M	61.9	309	IS1547-like transposase, pseudogene	MULP_035 (300)	100, 296	Transposase family	
MUDP_089	88090 – 89127	+	M	61.8	345	IS2404	IS2404 M. ulcerans (348)	99, 345	Transposase family	
MUDP_090	89689 – 90222	-	M	59.9	177	Conserved hypothetical protein	MULP_038 (177)	100, 177		
MUDP_091	90449 – 90658	-	M	60.0	69	Conserved hypothetical protein	MULP_039 (69)	100, 69		
MUDP_092	90926 – 91351	-	M	65.0	141	Conserved hypothetical protein	MULP_040 (141)	100, 141		
MUDP_093	91573 – 91776	+	M	64.7	67	Hypothetical protein				
MUDP_094	91932 – 92192	+	M	63.6	86	Conserved hypothetical protein	MULP_041 (86)	100, 86	One TMD	
MUDP_095	93145 - 93777	-	V	64.8	210	Conserved hypothetical protein	MUP023 (210) / MULP_042 (210)	99, 210		
MUDP_096	93774 - 94361	-	M	64.8	195	Conserved hypothetical protein	MULP_043 (195)	100, 195		
MUDP_097	95164 - 96201	-	M	61.8	345	IS2404	IS2404 (348)	99, 345	Transposase family	
MUDP_098	96524 - 98269	+	V	63.7	581	Transposase	MULP_060 (581)	99, 581	Transposase family	
MUDP_099	98352 - 99686	+	M	61.9	444	IS2606	IS2606 (444)	99, 444	Transposase mutator family	
MUDP_100	99885 - 100703	-	V	64.2	272	Transposase	MULP_047 (272)	99, 272	IS116/IS110/IS902 family transposase	
MUDP_101	100831 - 102576	-	V	63.7	581	Transposase	MULP_060 (581)	99, 581	Transposase family	
MUDP_102	102899 - 103936	+	M	61.8	345	IS2404	IS2404 (348)	99, 345	Transposase family	
MUDP_103	104175 - 104489	-	M	67.9	104	Hypothetical protein	MULP_050 (119)	100, 104		
